# Genetic crossroads of cardiovascular disease and its comorbidities: toward holistic therapeutic strategies

**DOI:** 10.1186/s12920-025-02279-1

**Published:** 2025-11-26

**Authors:** Binisha H. Mishra, Pashupati P. Mishra

**Affiliations:** Department of Clinical Chemistry, Fimlab Laboratories and Finnish Cardiovascular Research Center-Tampere, Faculty of Medicine and Health Technology, Tampere University, Tampere, 33520 Finland

**Keywords:** Cardiovascular diseases, Comorbidities, Genetic correlation, Multivariate GWAS, Genomic SEM, Pleiotropy, FinnGen

## Abstract

**Background:**

With increasing life expectancy, the prevalence of cardiovascular disease (CVD) accompanied by comorbidities is rising, presenting a growing challenge for healthcare systems. Understanding shared genetic factors underlying CVD and its comorbid conditions may facilitate the development of more effective strategies for prevention and treatment.

**Methods:**

In this study, we investigated genetic correlations between CVD and common comorbidities using genome-wide association study (GWAS) summary statistics from the FinnGen R12 release, comprising data from 500,000 Finnish individuals. Following standard quality control procedures, we examined 19 disease endpoints using linkage disequilibrium score regression (LDSC) to estimate heritability and pairwise genetic correlations. Disease traits with significant heritability (z-score ≥ 4) and Bonferroni-corrected significant correlations (adjusted *p* < 0.05) were selected for genomic structural equation modeling (Genomic SEM) to construct a latent genomic factor (LGF), representing shared genetic liability.

**Results:**

Out of the 19 diseases, four CVDs (transient ischemic attack, atrial fibrillation, myocardial infarction and heart failure) and seven comorbidities (type 2 diabetes, asthma, obesity, depression, chronic obstructive pulmonary disease, gingivitis and hypertension) showed statistically significant genetic correlations. A multivariate GWAS of the LGF identified 141 novel associated loci across 29 independent SNPs. These loci overlapped with 16 protein-coding genes, including *NPC1*, *TMEM106B*, *PTPN22*, *MAP2K5 and MSRA*, implicating them in the shared pathogenesis of CVD and its comorbidities.

**Conclusions:**

These findings underscore a shared genetic architecture between CVD and its comorbidities. We provide genetic evidence supporting the re-evaluation of these gene targets in the context of integrated, holistic and multi-disease treatment strategies.

**Supplementary Information:**

The online version contains supplementary material available at 10.1186/s12920-025-02279-1.

## Background

Cardiovascular diseases (CVDs) remain one of the most urgent global public health challenges, with significant implications for both human well-being and economic stability. In the European Union (EU), CVDs are the leading cause of mortality, accounting for approximately 32.4% of all deaths [1]. The economic burden is equally alarming, with an estimated annual cost of around €282 billion, driven by healthcare expenses, lost productivity, and informal care [2]. Despite global initiatives aimed at reducing their impact, progress in mitigating the burden of CVDs has been inadequate. At the same time, the prevalence of comorbid chronic conditions alongside CVDs continues to rise. This trend has led the European Commission to recognize multimorbidity, including comorbidities associated with CVD, as a key health priority, promoting awareness and fostering innovation in healthcare solutions to better address the growing complexities of managing multiple chronic conditions [3].

There is a growing consensus among public health experts and policymakers that healthcare systems must shift away from a narrow, single-disease focus toward a more holistic, multimorbidity-oriented approach. Effective management of complex cases involving CVD and its comorbidities requires a deeper understanding of how chronic diseases interact, how they jointly influence health outcomes, and how they affect treatment effectiveness and healthcare utilization. Managing CVD as a standalone condition often neglects the roles of co-occurring conditions such as diabetes, obesity, and depression, which can complicate care and reduce treatment efficacy.

It is crucial to move beyond clinical observations and investigate the underlying biological mechanisms that drive the co-occurrence of these conditions to develop more effective and targeted strategies. While previous studies have explored comorbid relationships between atherosclerosis and individual conditions such as osteoporosis [4], depression [5], lung disease [6], asthma [7], obesity and diabetes [8], comprehensive investigations that simultaneously consider multiple CVDs and their common comorbidities remain rare. One promising avenue for advancing this understanding is through the exploration of shared genetic architecture, which can reveal underlying biological connections between seemingly distinct CVD and its comorbidities.

A recent study by [9] investigated a similar multimorbidity concept using a genome-wide association study (GWAS) approach on cardiometabolic multimorbidity, defined as the co-occurrence of two or three cardiometabolic diseases. However, their study relied on traditional GWAS methods and did not analyze shared genetic architecture of the CVDs and their comorbidities using multivariate approaches. In contrast, the present study aims to assess the genetic correlations between CVD and its most prevalent comorbidities, identify shared genetic factors contributing to their co-occurrence and identify genetic variants associated with this common underlying genetic foundation utilizing advanced statistical methodologies. A deeper understanding of these shared genetic underpinnings of CVD and its comorbidities may uncover common biological pathways and guide the development of more integrated and effective shared strategies for their prevention, treatment, and management.

## Material and methods

This study was based on GWAS summary statistics of 19 clinical endpoints related to CVDs and their comorbidities from FinnGen data freeze 12 [10]. The original GWAS analyses were adjusted for key covariates, including sex, age, the first 10 principal components of genetic ancestry, genotyping chip version (FinnGen chip version 1 or 2), and genotyping batch. Clinical endpoints were defined using the Finnish adaptation of the International Classification of Diseases, 10th revision (ICD-10), as implemented in national health registers. Historical records using ICD-8 and ICD-9 were harmonized to ICD-10 definitions to ensure consistency across the full-time span of the registry data [10]. Full definitions are provided in Supplementary Table 1. The FinnGen study is a large-scale genomics initiative that has analyzed over 500,000 Finnish biobank samples and correlated genetic variation with health data to understand disease mechanisms and predispositions. The project is a collaboration between research organizations and biobanks within Finland and international industry partners. The number of cases and controls for each disease, along with the effective sample size (Neff), calculated as 4 times the product of the number of cases and controls, divided by their sum, i.e., Neff = *4* × *(cases​* × *controls)​/(cases* + *controls​​)*, to account for case–control imbalance, are presented in Table 1 [11]. Selection of GWASs on CVDs and their comorbidities was based on the comorbidities identified in [12], a comprehensive population-based study that systematically characterized major CVD comorbidities. Only those comorbidities that were available in FinnGen database were included. For classification purposes, we followed standard clinical and epidemiological definitions. Hypertension, although a major risk factor for CVD, is not classified as a CVD per se and was therefore categorized as a non-CVD comorbidity in our study. Similarly, pulmonary embolism, as a component of venous thromboembolism, primarily involves the venous rather than the arterial or cardiac systems and is typically considered distinct from classic CVDs. This classification was used to better capture shared genetic architecture between core CVD phenotypes and related but biologically or clinically distinct comorbidities. Since the same underlying cohort was used for all GWASs, the proportion of overlapping individuals between trait pairs varied from 7–76% depending on the phenotype definitions (Supplementary Table 2). Although the GWASs were derived from the same FinnGen cohort, differences in case definitions, exclusion criteria, and control group composition led to varying degrees of sample overlap between traits. This sample overlap was accounted for in the GenomicSEM modeling.

**Table 1:** Cardiovascular diseases and their associated comorbidities included in this study, with number of cases, controls, effective sample size and genome-wide significant loci identified by univariate genome-wide association studies (GWASs)

	**Diseases**	**Cases**	**Controls**	**Effective sample size**	**Significant loci**
Cardiovascular comorbidities					
1	Coronary atherosclerosis	63307	416171	219793.5	154
2	Transient ischemic attack	24948	453276	94586.05	2
3	Stroke	34110	450023	126827	10
4	Atrial fibrillation	63532	252810	203090.6	177
5	Myocardial infarction	31666	416171	117707.7	84
6	Heart failure	37653	462695	139277.9	18
7	Cardiomyopathy	7649	375343	29984.95	47
Non-cardiovascular comorbidities					
8	Type 2 diabetes	55418	405261	195005.7	272
9	Hypertension	154630	345634	427337.4	424
10	Asthma	47300	259839	160062.8	87
11	Metabolic dysfunction associated fatty liver disease	3504	496844	13917.84	7
12	Osteoporosis	10461	473264	40939.08	13
13	Obesity	31499	468693	118061.6	76
14	Depression	59333	434831	208836.2	45
15	Chronic obstructive pulmonary disease	24138	409070	91172.2	35
16	Epilepsy	15645	374605	60071.19	4
17	Pulmonary embolism	12762	486319	49742.65	33
18	Rheumatoid arthritis	16314	315115	62043.89	45
19	Gingivitis	137839	310260	381754.3	2

Quality control steps in the GWAS involved exclusion of participants with ambiguous gender, genotype missingness > 5%, excess heterozygosity (+ −4SD) and non-Finnish ancestry. SNPs with missingness > 2%, low HWE *p*-value < 1e-6 and minor allele count MAC < 3 were excluded [10].

Heritability for each disease and the genetic correlations between them were assessed using linkage disequilibrium score (LDSC) regression [11]. This study included only diseases with a heritability z-score ≥ 4, given that genetic correlation estimates below this threshold are typically characterized by high noise and limited reliability [13]. Genetic correlations with Bonferroni-adjusted *p*-values (adj.pval) < 0.05 were considered statistically significant.

A latent genetic factor (LGF) was constructed using genomic structural equation modeling (genomic SEM), based on the set of genetically correlated traits by leveraging the genetic covariance structure between them [14]. A multivariate GWAS of LGF was conducted using Genomic SEM to find shared genetic architecture of cardiovascular diseases and their comorbidities. Independent loci were identified using PLINK 2.0’s clumping procedure, with LD (linkage disequilibrium) threshold of r^2^ = 0.6 and a physical distance threshold of 1000 kilobases for clump formation [15]. The independent loci were annotated using SNPnexus, a web-based tool for genetic variant annotation using data from public repositories [16].

## Results

### Genetic correlation between CVDs and their comorbidities

**Fig. 1:**
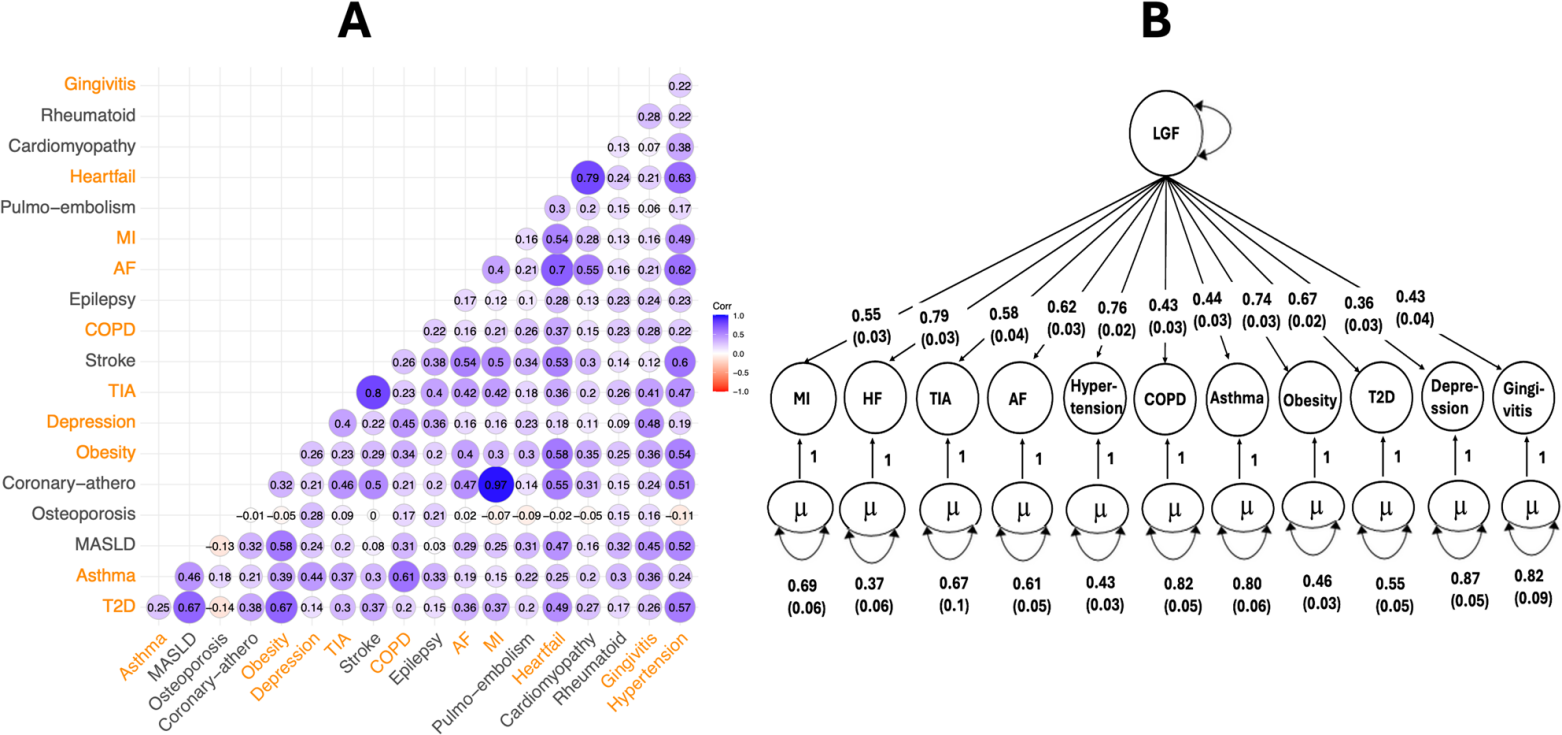
Genetic correlations and multivariate genetic factor model of cardiometabolic diseases. **A** SNP-based pairwise genetic correlations for the nineteen cardiometabolic disorders estimated using Linkage Disequilibrium Score (LDSC) regression. Trait names highlighted in orange on the x- and y-axes represent the subset of 11 traits selected for further analysis, based on statistically significant pairwise genetic correlations (Bonferroni-adjusted *p*-value < 0.05) among each other. Three exceptions are noted: gingivitis was not significantly correlated with heart failure and myocardial infarction, chronic obstructive pulmonary disease (COPD) was not significantly correlated with transient ischemic attack (TIA), and depression was not associated with heart failure. **B** Path diagram with standardized factor loadings in the hierarchical model estimated using Genomic SEM. The μ represents the residual variance that is not explained by the latent factor. *Abbreviations:* Pulmo-embolism, pulmonary embolism; MI, myocardial infarction; AF, atrial fibrillation and flutter; Coronary-athero, coronary atherosclerosis; MASLD, metabolic dysfunction-associated steatotic liver disease; T2D, type 2 diabetes mellitus; LGF, latent genetic factor, a surrogate variable for shared genetic foundation for these clinically distinct diseases

Out of 19 clinical endpoints related to CVD and their comorbidities, we considered genetic correlations with an adjusted *p*-value < 0.05 as statistically significant (Fig. 1A, Supplementary Table 3). In addition, we reported selected endpoints that did not reach this threshold, due to their broader relevance across multiple conditions. For instance, although gingivitis was not significantly correlated with heart failure and myocardial infarction, it showed significant genetic correlations with nine other conditions. Similarly, chronic obstructive pulmonary disease was not correlated with transient ischemic attack but was significantly correlated with all other endpoints. Depression was not correlated with heart failure but was significantly correlated with the remaining studied conditions. These findings indicate that, despite the lack of statistical significance for a few individual endpoints, these diseases remain important due to their consistent genetic correlations across most of the clinical outcomes. As a result, we selected 11 of the 19 diseases for further analysis of potential shared genetic etiology. These comprised four CVDs (heart failure, myocardial infarction, arterial fibrillation and transient ischemic attack) and seven comorbidities (type 2 diabetes, gingivitis, chronic obstructive pulmonary disease, depression, obesity, asthma and hypertension). Overall, the findings support a shared genetic foundation and etiology underlying these clinically distinct conditions.

### Shared genetic architecture of CVDs and their comorbidities

Using a multivariate GWAS of LGF underlying 11 genetically correlated CVDs and the comorbid conditions (Fig. 1B), we identified a total of 147 SNPs reaching genome-wide significance (*p*-value < 5 × 10^–8^). The LGF model showed a moderate fit, with a comparative fit index (CFI) of 0.83 and a standardized root mean square residual (SRMR) of 0.12. These fit indices indicate that the common factor captures a substantial portion of the shared genetic variance among the 11 traits, although some residual heterogeneity remains, suggesting that more complex factor structures may better represent the genetic architecture.

**Fig. 2:**
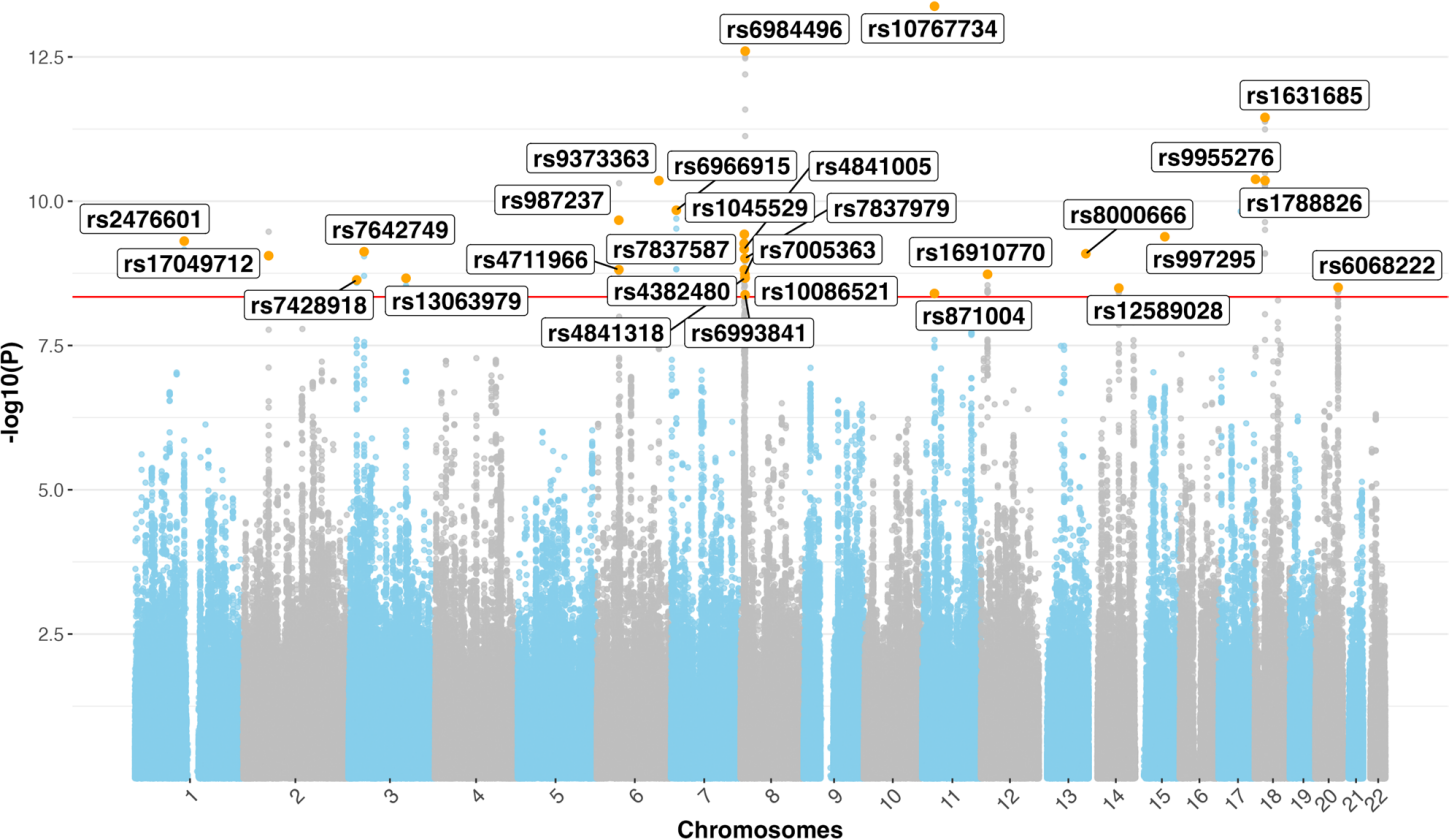
Manhattan plot of the multivariate genome-wide association study (GWAS) of the latent shared genetic factor (LGF) underlying cardiovascular diseases and their comorbidities. The *x*-axis shows genomic coordinates by chromosome, and the *y*-axis shows the − *log*_10_ of the unadjusted *p*-values for SNP associations with linkage disequilibrium score (LDSC) regression. Highlighted points indicate the 29 independent, novel SNPs identified through multivariate GWAS in this study. Associations were derived using GenomicSEM, leveraging linkage disequilibrium score (LDSC) regression

Of the 147 SNPs, 141 were not previously reported as genome-wide significant in any of the univariate GWASs for the 11 traits (Table 1). Of the 141 SNPs, 29 were identified as independent loci through PLINK-based clumping (Fig. 2) (Table 2). The 6 previously reported SNPs (*rs6741951, rs9856151, rs2397049, rs7831557, rs876954, rs1788817*), which reached significance in the multivariate model and showed associations across multiple traits mapped to genes *MSRA*, *NPC1*, *CPNE4*, and *RMC1*.

**Table 2:** List of 29 independent novel genetic variants with genomic location, *p*-values, and annotation, identified via a multivariate genome-wide association study (GWAS) of a latent genetic factor (LGF) representing genetically correlated cardiovascular diseases (CVDs) and their comorbidities. Also shown are the lowest p-values for each SNP across all individual CVD traits and across all comorbid traits from univariate GWAS

*rsID*	*Chromosome:position*	*P-value*	*Lowest p-value across all CVD traits*	*Lowest p-value across all comorbid traits*	*Gene*	*Type*	*Gene ontology – biological process*
rs10767734	11:28620834	4.2e-14	6.8e-05	2.7e-11	None	None	None
rs6984496	8:10938583	2.5e-13	2.9e-03	5.8e-17	*XKR6*	Protein coding	Apoptotic process
rs1631685	18:23554275	3.5e-12	1.4e-03	4.2e-12	*NPC1*	Protein coding	Cholesterol binding/signaling receptor activity
rs9955276	18:1839338	4.2e-11	1.3e-04	1.9e-12	None	None	None
rs1788826	18:23574060	4.4e-11	9.7e-04	1.0e-09	*NPC1*	Protein coding	Cholesterol binding/signaling receptor activity
rs9373363	6:142828906	4.4e-11	1.4e-05	2.0e-12	*HIVEP2*	Protein coding	Regulation of transcription
rs6966915	7:12226362	1.4e-10	1.1e-03	8.0e-10	*TMEM106B*	Protein coding	Dendrite morphogenesis/Lysosomal organization
rs987237	6:50835337	2.1e-10	3.1e-04	3.1e-19	*TFAP2B*	Protein coding	Glucose metabolic process/Regulation of transcription
rs1045529	8:9032588	3.7e-10	4.9e-03	1.2e-12	*ERI1*	Protein coding	RNA metabolism
rs997295	15:67724005	4.1e-10	5.3e-03	3.1e-13	*MAP2K5*	Protein coding	MAPK signaling cascade
rs2476601	1:113834946	4.9e-10	1.1e-05	7.0e-12	*PTPN22*	Protein coding	Immune system
rs7837587	8:8521482	5.4e-10	2.4e-02	1.5e-14	None	None	None
rs4841005	8:8643720	6.7e-10	1.0e-02	3.6e-13	None	None	None
rs7642749	3:35627397	7.5e-10	2.7e-03	1.9e-08	None	None	None
rs8000666	13:107712256	8.1e-10	6.9e-02	3.6e-02	*FAM155A*	Protein coding	Calcium channel activity
rs17049712	2:58734001	8.8e-10	1.4e-01	2.9e-12	*LINC01122*	lincRNA	None
rs7837979	8:10341024	9.8e-10	1.4e-03	4.0e-12	*MSRA*	Protein coding	Methionine metabolic process/Response to oxidative stress
rs4382480	8:8863963	1.5e-09	3.1e-04	3.5e-12	*MFHAS1*	Protein coding	Inflammatory response
rs4711966	6:50996064	1.5e-09	7.8e-06	6.3e-10	None	None	None
rs16910770	12:15352797	1.8e-09	1.4e-03	9.4e-11	*PTPRO*	Protein coding	Regulation of glomerular filtration
rs7005363	8:10426238	1.9e-09	3.0e-03	3.3e-16	*MSRA*	Protein coding	Methionine metabolic process/Response to oxidative stress
rs10086521	8:10926259	2.1e-09	1.8e-03	3.7e-09	*XKR6*	Protein coding	Apoptotic process
rs4841318	8:10329690	2.1e-09	1.4e-02	3.4e-12	*MSRA*	Protein coding	Methionine metabolic process/Response to oxidative stress
rs13063979	3:131845897	2.2e-09	3.3e-04	8.9e-09	*CPNE4*	Protein coding	Cellular response to calcium ion
rs7428918	3:18782806	2.3e-09	2.1e-04	1.3e-09	*SATB1-AS1*	Processed transcript	None
rs6068222	20:52541802	3.1e-09	5.4e-03	3.8e-09	*LINC01524*	lincRNA	None
rs12589028	14:69076025	3.2e-09	1.8e-03	1.1e-08	*DCAF5*	Protein coding	Protein ubiquitination/Fatty acid biosynthetic process
rs871004	11:28490911	4.0e-09	3.1e-03	2.2e-14	*METTL15*	Protein coding	Methylation
rs6993841	8:10775174	4.2e-09	1.2e-03	2.3e-12	*PINX1*	Protein coding	Telomere maintenance

To aid interpretation of multivariate results, for each SNP, we report the lowest *p*-value among CVD traits and the lowest *p*-value among comorbid traits in Table 2. We report the marginal associations (β, SE, and *p*-values) of all 29 genome-wide significant SNPs across the 11 input traits in Supplementary Table 4. This table also includes the heterogeneity Q-values, which indicate whether the SNP effects are consistent across traits or driven predominantly by one or a few. Additionally, Supplementary Table 5 presents the standardized factor loadings, standard errors, and *p*-values for each trait from the LGF model. This allows differentiation between SNPs likely to be truly pleiotropic and those predominantly driven by one trait domain. Notably, 10 SNPs exhibited Q *p*-values < 0.001, indicating that their effects were primarily driven by a subset of traits, whereas the remaining loci showed more consistent effects across multiple traits. These patterns highlight the importance of considering multivariate significance alongside marginal associations and Q_SNP statistics.

These 29 SNPs mapped to 16 protein-coding genes involved in several key biological processes associated with CVDs and their comorbidities, including *NPC1*, *TMEM106B*, *PTPN22*, *MAP2K5*, and *MSRA***,** which are further discussed for their pleiotropic effects and potential therapeutic relevance. Importantly, several of the 29 independent SNPs had only modest or nominal associations in individual univariate GWAS, illustrating how the multivariate LGF approach captures variants contributing to shared genetic liability that may be missed when examining traits individually. Functional annotation of these loci revealed that these included fundamental cellular functions such as metal ion binding, methyltransferase activity, and protein metabolism; key metabolic processes like the regulation of fatty acid biosynthesis and LDL clearance; as well as pathways involved in calcium ion import across the plasma membrane, signal transduction, immune system function, dendrite morphogenesis, lysosomal trafficking and apoptosis (Table 2). Collectively, these results provide evidence for a shared genetic architecture underpinning clinically distinct yet genetically correlated cardiovascular and comorbid conditions.

## Discussion

In this study, we utilized multivariate genomic approaches to identify novel genetic loci associated with a LGF underlying 11 genetically correlated CVDs and related comorbidities, using GWAS summary statistics from the wide FinnGen database. These 11 conditions were selected from an initial set of 19 based on significant genetic correlations, reflecting a shared genetic architecture. These findings suggest that the co-occurrence of CVDs and their comorbidities may stem from overlapping pleiotropic genetic mechanisms, rather than solely from shared environmental or behavioral risk factors. Through multivariate GWAS of the LGF, our analysis uncovered genetic loci not previously implicated in individual GWASs of CVDs, underscoring the utility of modeling shared genetic foundation and liability. These findings highlight pleiotropic mechanisms that may contribute to both cardiovascular pathology and its related comorbidities, thereby offering promising avenues for future genetic, biological, and therapeutic research.

To further support the interpretation of these multivariate GWAS results, we examined the marginal associations of all 29 genome-wide significant SNPs across the 11 included traits (Supplementary Table 4). This allowed us to assess whether these SNPs reflect truly pleiotropic effects or are predominantly driven by associations with specific subsets of traits (for example, only CVDs or only comorbidities). We also report heterogeneity Q-values derived from the GenomicSEM framework, which help quantify the extent to which SNP effects diverge across traits. Additionally, the standardized factor loadings, standard errors, and *p*-values for the 11 traits on the latent genetic factor are presented in Supplementary Table 5. These loadings ranged from 0.36 to 0.79, with 7 of the 11 traits loading ≥ 0.5, suggesting that the latent factor captures a robust and broadly shared genetic architecture spanning both cardiovascular and comorbid conditions.

While all 29 SNPs identified in the multivariate GWAS reached genome-wide significance for the LGF and are therefore relevant to the shared genetic architecture of CVD and comorbid traits, inspection of marginal associations and Q_SNP heterogeneity statistics provides critical context for interpretation. Multivariate significance does not require strong univariate effects in every trait; rather, GenomicSEM leverages cross-trait genetic covariance to detect variants that might be missed in univariate analyses. By reporting the lowest p-values across CVDs and comorbid traits (Table 2) alongside heterogeneity measures, we distinguish loci that are likely pleiotropic across both domains from those predominantly driven by one set of traits.

Functional annotation revealed that many of the identified genes are involved in biological processes central to cardiovascular and metabolic health, including lipid metabolism, vascular integrity, mitochondrial function, inflammation, and oxidative stress. Among the most compelling findings is the association of a SNP within *NPC1* (Niemann-Pick disease, type C1), a gene essential for intracellular cholesterol transport and lipid homeostasis [17]. Dysregulation of NPC1 has been implicated in atherosclerosis [18], and its association with the LGF in our study reinforces its role in lipid trafficking as a shared pathogenic mechanism across genetically correlated CVDs. *NPC1* showed strong multivariate significance, suggestive associations with CVD traits (*p*-value ≈ 1e-03), and strong associations with comorbid trait (*p*-value ≤ 1.0e-09), indicating that its LGF contribution is largely comorbid trait-driven, consistent with its known role in cardiometabolic disease.

*MSRA* (methionine sulfoxide reductase A) was also genome-wide significant for the LGF. Known for its antioxidant role in repairing oxidized methionine residues [19], *MSRA* has been linked to improved lipid metabolism and atherosclerotic protection in animal models [20] and to neuropsychiatric phenotypes in human cohorts [21, 22]. In our dataset, *MSRA* showed modest CVD associations (lowest *p*-value = 1.4e-03) but stronger evidence in comorbid traits (*p*-value ≤ 4.0e-12), suggesting it is more comorbid trait-driven. Nonetheless, its role in oxidative stress defense and prior pleiotropic links underscore its relevance to both cardiovascular and systemic health.

For *TMEM106B* (Transmembrane Protein 106B), a locus previously linked to both depression and coronary artery disease [23], we observed genome-wide significance in the LGF, strong association with comorbid traits (*p*-value = 8.0e-10) but only marginal evidence in CVD traits (*p*-value = 1.1e-03). This indicates that in our dataset, the locus is primarily comorbid trait-driven rather than broadly pleiotropic, although prior evidence suggests potential cross-system mechanisms.

*PTPN22* (Protein Tyrosine Phosphatase Non-Receptor Type 22), a well-known immune regulator, has been implicated in autoimmune disease and inflammation. Recent studies suggest roles in calcific aortic valve disease pathogenesis [24], highlighting therapeutic potential through modulation of immune activity. In our results, *PTPN22* showed strong associations with both CVD (*p*-value = 1.1e-05) as well as with comorbid traits (*p*-value = 7.0e-12).

*MAP2K5*, a component of the *MAPK/ERK5* signaling pathway that influences endothelial dysfunction and atherosclerosis [25, 26], reached genome-wide significance in the LGF and strong comorbid signal (*p*-value = 3.1e-13) but showed only nominal evidence in CVD traits (*p*-value = 5.3e-03). Thus, *MAP2K5*’s contribution appears modest and primarily comorbid trait-specific, though its pathway-level functions remain biologically relevant.

Other notable genes included *XKR6* (XK-related protein 6), implicated in apoptotic processes central to atherosclerosis [27], and *ERI1* (exoribonuclease 1), which may influence vascular remodeling through RNA metabolism [28]. *PTPRO* (protein tyrosine phosphatase receptor type O) points to the renal–cardiovascular axis via its regulation of glomerular filtration [29]. *METTL15* (Methyltransferase Like 15), involved in mitochondrial RNA methylation [30], underscores the importance of mitochondrial integrity in cardiovascular disease. Genes such as *HIVEP2* (human immunodeficiency virus type I enhancer binding protein) and *CPNE4* (copine 4), though lacking direct CVD associations, contribute to neurodevelopmental and metabolic processes [31, 32], which may intersect with cardiometabolic risk.

To assess the external validity of our results, we cross-referenced them with prior GWAS findings. This analysis showed that 14 SNPs had been previously associated with relevant traits in independent cohorts, including type 1 and type 2 diabetes, body mass index, waist-hip ratio, insomnia, neuroticism, rheumatoid arthritis, and metabolite levels. These associations are presented in Supplementary Table 6. Although we did not aim to replicate individual SNP associations, the presence of such overlaps across diverse phenotypes supports the biological plausibility and pleiotropic nature of the identified loci.

There were certain limitations in our study. First, this study is based on GWAS summary-level data. However, the use of summary statistics from FinnGen, a large, well-characterized population cohort, allows for harmonized, high-powered analyses across multiple traits. GenomicSEM is specifically designed for such data and has been widely validated. While individual-level data would enable more flexible modeling, access to such data at comparable scale is often not feasible.

Also, effective sample size for each clinical endpoint varied, ranging from 13917.84 for MAFLD to 427337.4 for hypertension and the GWASs of the selected clinical endpoints available in FinnGen database had sample overlap ranging from 7–76%. The varying sample size and overlaps may have impacted our study findings. The moderate fit indices (CFI = 0.83, SRMR = 0.12) for the LGF model indicate that while the common factor effectively captures shared genetic influences across CVD and comorbid traits, the genetic architecture is likely more complex. Future work could explore multi-factor models or alternative structures to better characterize distinct pleiotropic patterns and trait-specific effects. Also, sex-stratified analysis was not possible due to the use of GWAS summary data as input. Therefore, further research should investigate how shared genetic risks differ by sex. Lastly, all samples were primarily of European ancestry; therefore, our findings are relevant only for European-ancestry populations.

## Conclusions

In conclusion, our multivariate GWAS identified 29 loci contributing to a LGF shared across CVD and comorbid traits. While all 29 loci are valid discoveries under the GenomicSEM framework, marginal associations and Q_SNP heterogeneity reveal heterogeneity in how individual loci act. Based on marginal *p*-values (Table 2), the majority of loci are primarily comorbid trait**-**driven in our data. The implicated pathways converge on lipid metabolism, mitochondrial function, inflammation, apoptosis, RNA regulation and neurovascular signaling. These genes therefore remain promising candidates for follow-up, but their prioritization for therapeutic development should reflect whether they are pleiotropic or domain specific as indicated by the marginal and heterogeneity data.

## Supplementary Information

Supplementary Material 1: Table S1: List of 19 diseases including cardiovascular diseasesand their comorbidities and their definitions. Table S2: Percentage of sample overlap between FinnGen genome-wide association studiesof the 19 diseases. Table S3: Statistical significanceof the genetic correlation among the 19 cardiovascular diseases and their comorbidities as estimated using Linkage disequilibrium scoreregression. Table S4: Summary of 29 SNPs identified from multitrait GWAS via GenomicSEM, including original GWAS betas, standard errors, *p*-values, alternate allele frequency, alternate allele frequency among cases, alternate allele frequency among controlsand Q-SNP heterogeneity statistics. Table S5: Standardized factor loadings and standard errors for individual traits in the GenomicSEM model of the genetic latent factor. Table S6: Previously reported GWAS associations for 29 independent SNPs supporting external validity of multivariate GWAS findings.

## Data Availability

Summary statistics from the FinnGen study are made openly available to the research community following a short embargo period. Access to individual-level data is restricted to research partners and provided within a secure analysis environment, ensuring compliance with data protection regulations and safeguarding participant privacy. Further information on data access procedures is available through the FinnGen website. Codes used in the analysis are available on GenomicSEM wiki page ([https://github.com/GenomicSEM/GenomicSEM/wiki](https://github.com/GenomicSEM/GenomicSEM/wiki)).

## Abbreviations

CVD: Cardiovascular disease
GWAS: Genome-wide association study
LDSC: Linkage disequilibrium score
LGF: Latent genetic factor
ICD: International classification of diseases
SD: Standard deviation
HWE: Hardy-Weinberg equilibrium
MAC: Minor allele count
GenomicSEM: Genomic structural equation modelling
CFI: Comparative fit index
SRMR: Standardized root mean square residual
